# Realization of an Ultra-thin Metasurface to Facilitate Wide Bandwidth, Wide Angle Beam Scanning

**DOI:** 10.1038/s41598-018-23288-4

**Published:** 2018-03-19

**Authors:** Alpha O. Bah, Pei-Yuan Qin, Richard W. Ziolkowski, Qiang Cheng, Y. Jay Guo

**Affiliations:** 1 Global Big Data Technologies Centre, University of Technology Sydney, New South Wales, 2007 Australia; 2 Southest University, Nanjing, Jiangsu China

## Abstract

A wide bandwidth, ultra-thin, metasurface is reported that facilitates wide angle beam scanning. Each unit cell of the metasurface contains a multi-resonant, strongly-coupled unequal arm Jerusalem cross element. This element consists of two bent-arm, orthogonal, capacitively loaded strips. The wide bandwidth of the metasurface is achieved by taking advantage of the strong coupling within and between its multi-resonant elements. A prototype of the proposed metasurface has been fabricated and measured. The design concept has been validated by the measured results. The proposed metasurface is able to alleviate the well-known problem of impedance mismatch caused by mutual coupling when the main beam of an array is scanned. In order to validate the wideband and wide scanning ability of the proposed metasurface, it is integrated with a wideband antenna array as a wide angle impedance matching element. The metasurface-array combination facilitates wide angle scanning over a 6:1 impedance bandwidth without the need for bulky dielectrics or multi-layered structures.

## Introduction

The material properties: permittivity and permeability, can be used to classify all materials including metamaterials (MTMs)^1^. MTMs are engineered materials composed of sub-wavelength unit cells with unique properties that may not usually be available in nature^2–6^. Modification of the MTM geometry can be used to tune its electric and or magnetic response thereby producing tailored values of permittivity or permeability. The resulting modification of the effective material properties may also affect the MTM’s transmission, reflection, absorption, and coupling capabilities^7^. MTM applications include negative index materials^8–11^, near zero index materials^12,13^, gradient index materials^14^, and electromagnetic cloaking^15–17^.

Due to the thickness and weight required to achieve the desired MTM properties, researchers have realized the efficacy of planar, two-dimensional equivalents known as metasurfaces (MSs). Metasurfaces are usually planar, easier to fabricate, and weigh much less when compared to 3D MTM structures. They are also less lossy due to their reduced size^18^. Some applications of MSs include: angular-independent surfaces^19–21^, absorbers^22–24^, radar cross section reduction surfaces^25^, wave front shapers^26^, ultra-thin transmission-type metalenses^27–30^, and wide angle impedance matching layers (WAIMs)^31,32^. WAIMs are usually employed to minimize the amount of scan loss in phased array antennas. This scan loss is caused by the variations in the array’s active impedance that arise from changes in the mutual coupling between the radiating elements as the scan angle and frequency change^33^.

Several WAIM schemes have been introduced and used over the years. The earliest techniques involved using thin high-dielectric-constant superstrates in front of an array aperture^34^ or dielectric slabs adjacent to the array aperture^35,36^. The above WAIM schemes either improve scanning in one plane at the expense of the others^34^, or increase the chance of array blindness^35,36^. These WAIM techniques were also limited to single frequency or narrow bandwidth operations.

To improve the bandwidth of WAIM structures, multi-layered MTM^37,38^ structures and dielectrics^39^ have been used. Unfortunately, the overall volume and weight of the array is also increased. To enable wide angle scanning without increasing the volume and weight of antenna arrays, MSs have been employed. The anisotropic MS-WAIM (henceforth referred to as MS only for brevity) in ref.^31^ provided an improved match for an array of open-ended circular waveguides to free space over several angles. However, only the scanning results for the H-plane were presented; the associated E-plane and D-plane (diagonal plane of a radiating aperture) results were not reported. Moreover, wide angle scanning over a narrow bandwidth was the focus of the design, which was achieved over a 3.3% bandwidth. The works reported in ref.^34^ and ref.^31^ were extended in ref.^32^. The dielectric slab was replaced with an ultra-thin metasurface composed of subwavelength split-ring resonators (SRRs) for an improved scan in the H and D-planes. Simulation results showed that wide angle scanning was achieved over a 20% impedance bandwidth.

While there has been some advancement on WAIM metasurfaces, most of the past work was focused on wide angle scanning over narrow bandwidths^31,32^. In this paper, we propose a new ultra-thin MS that can achieve both wideband operation and wide angle scanning. The MS is composed of strongly-coupled (termed tightly-coupled in the antenna literature) unequal arm Jerusalem cross (TC-UAJC) elements. The TC-UAJC is an evolved version of the Jerusalem cross^40^ and its derivatives^41,42^. The wide bandwidth is obtained by taking advantage of the tight coupling within and between the multi-resonant elements. The band of operation is located much lower than the MS’s resonance frequency to avoid the associated highly dispersive and lossy regions^31^. These losses are due to the total power absorbed by the MS near its resonance frequency. At frequencies near its resonance, the field concentration per unit cell in the metallic layers of the structure is increased, which in turn leads to an increase in the resistive heating^43^. This MS is fairly insensitive to the changing phases of the signals incident upon it over a wide bandwidth. As a result, it is able to maintain its performance for scanning angles of up to 70^ο^ from the normal to the MS for both transverse magnetic (TM) and transverse electric (TE) polarized incident waves. The metasurface is integrated with a tightly coupled antenna array to validate its feasibility. It is shown that the metasurface-array combination provides improved scanning along the E (72^ο^), Η (80^ο^), and D (79^ο^) planes over a 6:1 impedance bandwidth without the need for bulky dielectrics or multi-layered structures, resulting in a light-weight antenna system with reduced profiles.

## Results

### Unit cell structure and operation

**Figure 1:**
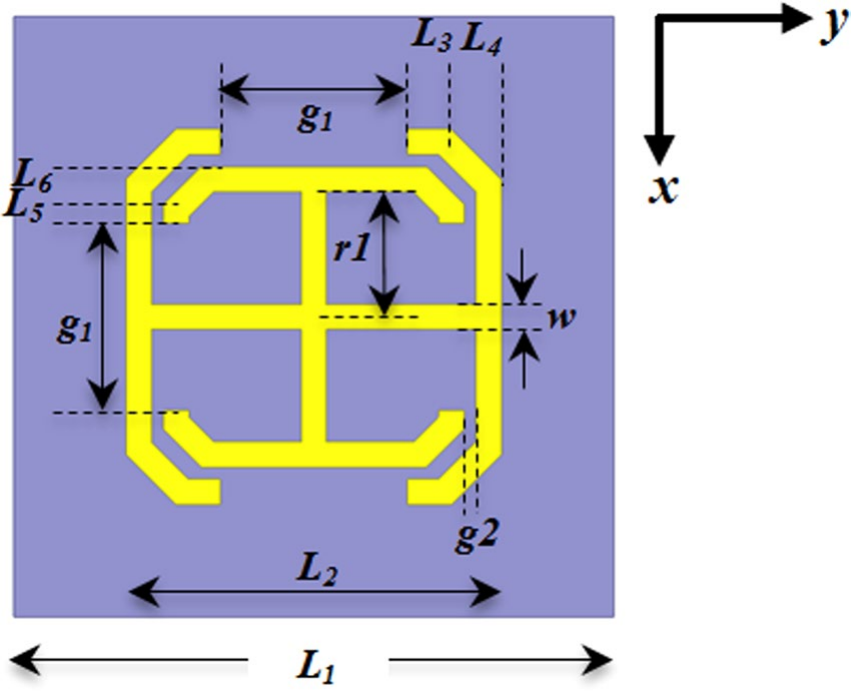
Top view of the MS unit cell geometry. The optimized unit cell dimensions for the MS are: w = 0.2 mm, H_sub_ = 0.254 mm, g_1_ = 1.5 mm, g_2_ = 0.1 mm, L_1_ = 4.8 mm, L_2_ = 3.0 mm, L_3_ = 0.35 mm, L_4_ = 0.4 mm, L_5_ = 0.15 mm, L_6_ = 0.3 mm, and r_1_ = 1.0 mm.

The unit cell configuration of the MS is depicted in Fig. 1. The metallic pieces (in yellow) are etched on the top surface of a dielectric substrate. The TC-UAJC element consists of two orthogonal, bent-arm capacitively-loaded-strips. They are tuned for the desired operating frequencies. These arm segments provide inductance; the gaps between the extremities of its bent arms provide capacitance.

In order to minimize the variation of the array active reflection coefficients at wider scan angles, the MS introduces a capacitive reactance below its resonance frequency point to counteract the effects of the array’s inductively reactive ground plane. The MS serves as a wideband impedance transformer between the array aperture and free space. To ensure a low loss MS, its operational bandwidth is located way below its resonance frequency.

The TC-UAJC element presented in this paper uses three main techniques to achieve optimal transmission and minimal reflection over a wide bandwidth below its resonance frequencies. Firstly, tightly coupled elements are used to increase their inter-element capacitance. It should be noted that smaller inter-element spacing produces large bandwidths but can lower the resonance frequency if the resulting capacitance is excessive^44^. The smaller inter element spacing combined with the subwavelength nature of the MS elements allows field propagation to neighbouring elements and the elimination of impedance variations to give rise to large bandwidths^40^. In the extreme case when the elements are very small and tightly packed, the current distribution across the MS approaches the Wheeler uniform current sheet concept^45^. The reduction in resonance frequency comes as a result of the well-known relationship, $\omega =1/\sqrt{LC}$. Secondly, the horizontal and vertical arms of the TC-UAJC element are tightly coupled to each other for increased intra-element capacitance and thin traces are used for increased inductance, thereby enabling both compactness and an inherently wideband element^46^. Thirdly, the constituent parts of the element provide closely spaced multiple resonances^47^ which combine to produce a wide bandwidth.

### Metasurface design and analysis

The proposed MS is designed using the commercially available ANSYS High Frequency Structure Simulator, ANSYS-HFSS^48^. A single-sided MS was simulated and an initial optimization was carried out to obtain the optimum transmission and reflection values. A prototype of the optimized MS was fabricated based on these optimized values and was tested to verify its operation. The effective medium parameters of the MS were also extracted from the MS S-parameters using MATLAB^49^ and the method described in ref.^50^. To demonstrate the WAIM capability of this MS across a wide frequency range, it is then integrated with a tightly coupled antenna array (TCAA) in an HFSS model for a final optimization. The antenna array consists of a set of tightly coupled dipole antennas^39^ with less than 0.4 λ spacing between them.

#### Single Sided Metasurface

The single sided MS was modeled using master-slave periodic boundary conditions in the x and y directions of the unit cell of Fig. 1. The periodic boundaries enforce a linearly progressive phase shift between the master and slave boundary pairs with uniform amplitude to enable beam scanning in the required directions. Two Floquet ports were used to illuminate both sides of the MS, one located at the top face and the other at the bottom face of the model’s air box. The length of the airbox was chosen to ensure that all higher order modes other than the two propagating zeroth-order Floquet modes experience at least 70 dB of attenuation.

**Figure 2:**
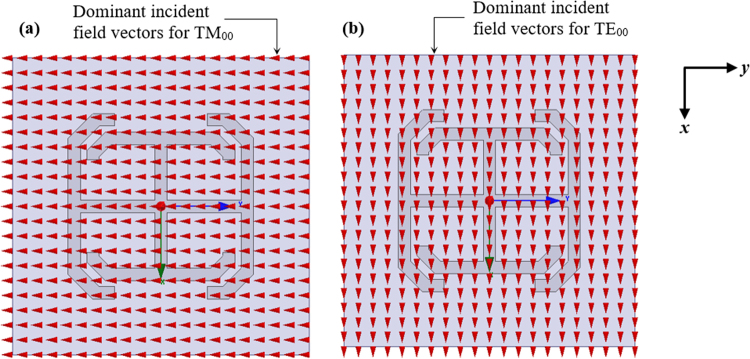
Top view of the unit cell. (**a**) The TM_00_ floquet mode fields. (**b**) The TE_00_ Floquet mode fields at the face of the top Floquet port.

The copper-cladded Rogers RT/Duroid^TM^ 5880 substrate with a relative dielectric constant of 2.2 and a height of *H*_*sub*_ = 0.254 *mm* was selected. The TC-UAJC element was etched on the 17 μm thick copper sheet on its upper surface. The HFSS solution frequency was selected to be 20 GHz for a (0.5–20) GHz frequency sweep. The maximum scan volume was θ = 70°. To minimize the simulation time and the amount of discretization, the metals in the HFSS model were taken to be perfect electric conductors (PECs). The zeroth-order Floquet modes, TM_00_ and TE_00_, were used for scanning in the *y-z* and *x-z* planes respectively as shown in Fig. 2. The electric field of the TM_00_ Floquet mode is parallel to the plane of incidence (along the y-direction) and the electric field of the TE_00_ Floquet mode is perpendicular to the plane of incidence (along the x-direction).

**Figure 3:**
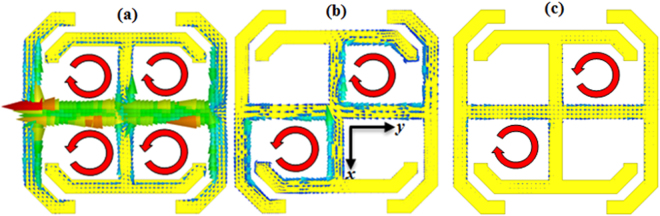
The surface current densities on the MS for normal incidence showing circulating current loops. (**a**) TM reflection resonance at 15.8 GHz. (**b**) TE reflection resonance at 19.5 GHz. (**c**) TE transmission resonance at 19.7 GHz.

To determine the orientation of an antenna with respect to the MS element, the surface current distribution on the element is studied. The resulting surface current densities on the MS element for the corresponding TE and TM reflection (i.e., |S11|→0) and transmission (i.e., |S21|→0) resonances for normal incidence are shown in Fig. 3. For the TE reflection and transmission resonance cases, the MS is only weakly excited. For the TM reflection resonance case on the other hand, the MS is strongly excited with opposite sense circulating current loops. The circulating surface currents flow in equal and opposite directions in adjacent regions of the MS indicating that the magnetic fields created cancel out each other leaving no net magnetic response. Current flow is maximum along the *y*-direction suggesting that the optimum radiator-MS coupling is achieved when a radiator’s E-plane is aligned with the *y*-axis of the MS.

For a MS to act as an efficient wideband WAIM, it must be able to fulfil certain basic criteria. Firstly, the intended operational frequencies of the antenna array should be considerably lower than the resonance frequencies of the MS to avoid the highly dispersive and lossy regions^31^. Secondly, it should have little effect on the phases of the signals incident upon it during scan across the whole band of interest in order to facilitate the same output response. Thirdly, it should be thin and light weight to help reduce the volume and profile of the antenna array.

**Figure 4:**
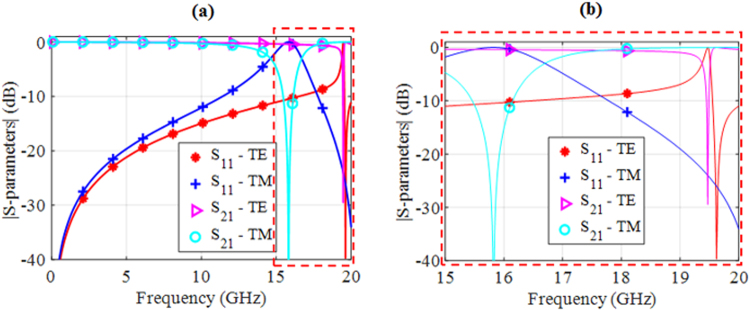
Magnitudes of the S-parameters for normal incidence. (**a**) TE and TM excitations of the MS. (**b**) An expanded view showing the first TM reflection resonance at 15.8 GHz and the first TE reflection resonance at 19.5 GHz. A TE transmission resonance can also be seen at 19.7 GHz.

In Fig. 4, we show with the simulated scattering parameters (S-parameters) at broadside, that the reflection resonance frequencies of the MS are above 15.8 GHz, which is much higher than the intended operational frequencies of the antenna array (below 5.0 GHz). The return loss for both the TE and TM polarizations is greater than 20 dB across the frequency band of interest and, hence, the insertion loss is virtually zero. Due to the unequal lengths of the two arms of the TC-UAJC element, they exhibit slightly different reflection resonance frequencies when illuminated by the TM and TE incident waves. Each resonance frequency is directly proportional to the unwound length, L, of the TC-UAJC element^51^.The first reflection resonance occurs at 15.8 GHz for the TM excitation when the electric field is along the longer arm; the second one occurs at 19.5 GHz for the TE excitation when it is along the shorter arm.

**Figure 5:**
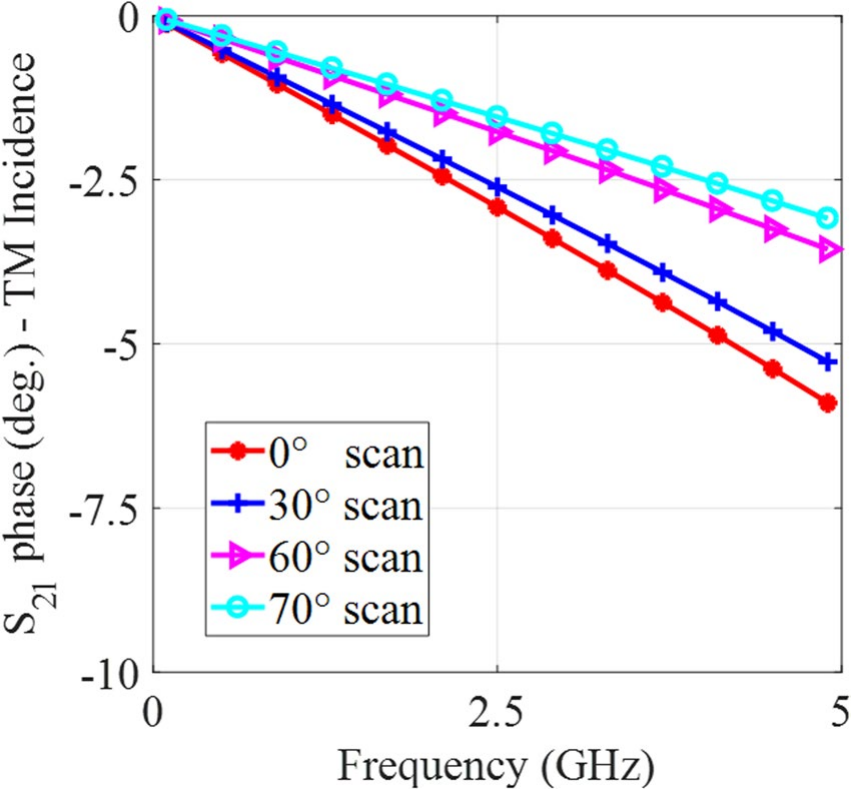
Transmission phase variation with frequency for the TM polarized incident fields for various angles of incidence.

The transmission phase variation with frequency for the TM polarized incident fields for various angles of incidence is shown in Fig. 5. The phase varies by only 3° for a 0°–70° change in the incident angle. This small change in phase implies that the MS will hardly alter the phase of electromagnetic waves traversing it^22^. The resulting MS is very thin and light weight. It is only 0.254 mm thick.

#### Design Parameter Studies

One of the major goals was to make the reflection magnitude and transmission phase across the frequency band of interest as small as possible without having an adverse impact on the bandwidth of the MS. Consequently, several of the design parameters were studied to determine the values that produced the minimum reflection magnitude and transmission phase. It was found that the parameters g_1_, g_2_, r_1_, and H_sub_ have the most impact on the MS performance.

**Figure 6:**
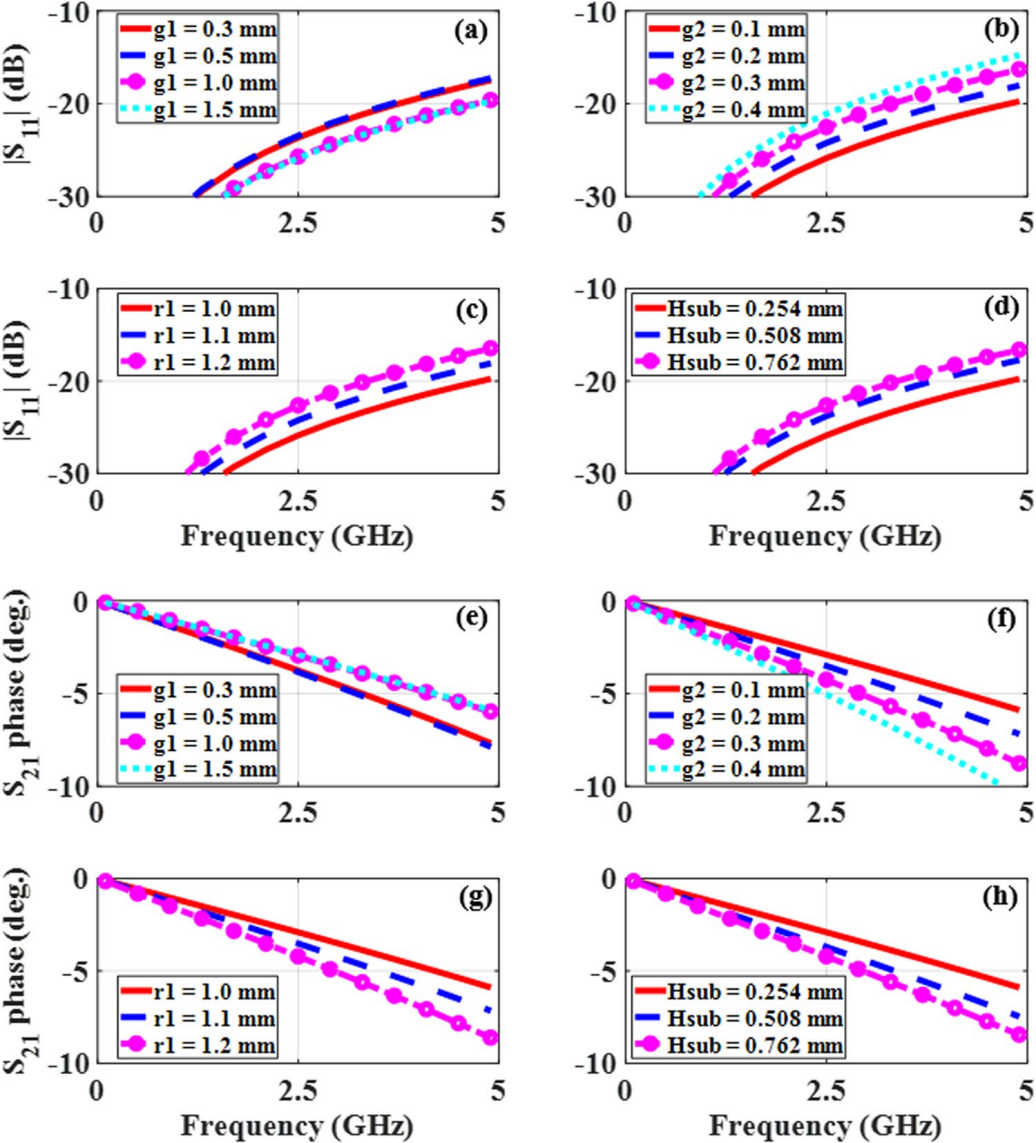
The effects of various MS design parameters on the reflection magnitude (**a**–**d**) and transmission phase (**e**–**h**) as functions of the excitation frequency for the TM incidence case. (**a**) and (**e**) g1. (**b**) and (**f**) g2. (**c**) and (**g**) r1. (**d**) and (**h**) Hsub.

The effects of g_1_ on the reflection magnitude and transmission phase values are shown in Fig. 6a,e, respectively. Increasing g_1_ reduces both the reflection magnitude and transmission phase values. However, further increase beyond 1 mm has negligible impact. The effects of g_2_ are shown in Fig. 6b,f. Decreasing g_2_ gives decreasing values for both the magnitude and phase. The value of g_2_ is limited by manufacturing tolerances so it has been confined to a minimum value of 0.1 mm. The parameters g_1_ and g_2_ control the amount of intra-element coupling. Smaller values of g_2_ indicate a tighter coupling between the horizontal and vertical arms of the TC-UAJC resulting in a large coupling capacitance. When this coupling capacitance is combined with the high loop inductance produced by the thin traces of the structure, the element bandwidth is enhanced.

Figure 6c,g show the effects of r_1_. Decreasing r_1_ gives decreasing values for both the magnitude and phase. The minimum value of r_1_ has been restricted to 1 mm for ease of fabrication. The resonance frequency of the unit cell is essentially determined by r_1_. Smaller values of r_1_ lead to smaller elements with correspondingly higher resonance frequencies, creating a wider separation between the frequency band of interest and the element resonance frequency. The result from this wide separation is a smaller transmission phase variation with respect to the scan angle. The inter-element coupling is controlled by the values of r_1_.

The effects of *H*_*sub*_ are shown in Fig. 6d,h. Decreasing *H*_*sub*_ gives decreasing values for both the magnitude and phase of the S-parameters. Thinner substrates, i.e., smaller *H*_*sub*_, ensure higher transmission and minimal reflection of the incident signal. From all of these reflection magnitude and transmission phase plots given in Fig. 6, the optimum values are: g_1_ = 1.5 mm, g_2_ = 0.1 mm, r1 = 1.0 mm and Hsub = 0.254 mm.

#### Parameter Extraction

**Figure 7:**
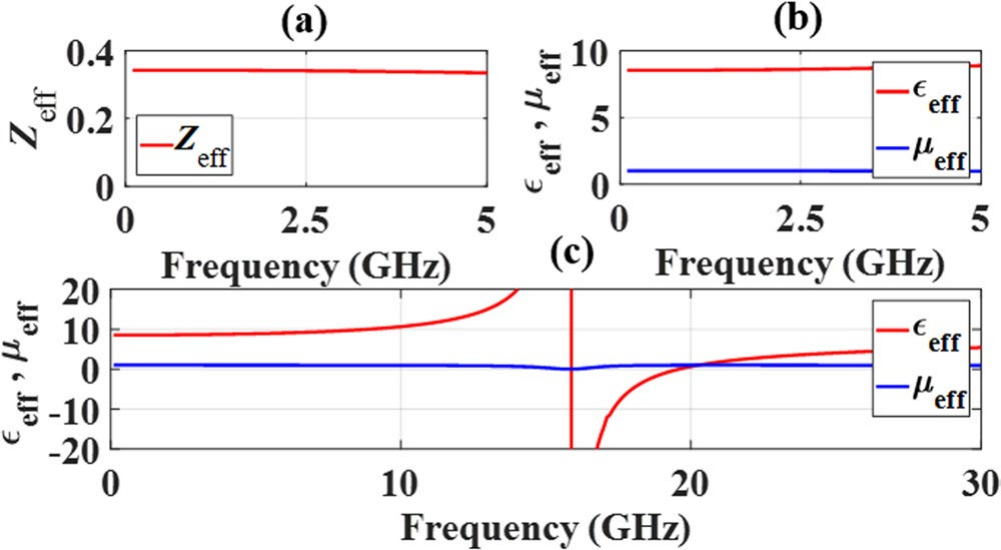
Extracted parameters of the MS for the TM excitation. (**a**) Effective impedance. (**b**) Effective permittivity and effective permeability at the frequencies of interest and (**c**) across the whole band showing TM resonance at 15.8 GHz.

To further characterize the MS’s response, it is of interest to determine its effective material and wave parameters. The method described in ref.^50^ was used to extract the effective wave impedance Z_eff_, the effective dielectric constant ε_eff_, and the effective permeability μ_eff_ from the S-parameters. These wave and medium parameters are shown in Fig. 7. During the extraction process, the MS was assumed to be homogeneous with an effective thickness equal to that of the substrate upon which the TC-UAJC element resides. Homogenization is justified due to the sub-wavelength nature of the MS inclusions. Owing to the MS’s very thin nature in the direction of propagation, the extraction process was expected to proceed smoothly. Moreover, its thinness led to virtually the same results relative to each port of the unit cell. In order to obtain more complete extraction results, the MS was simulated up to 30 GHz for the TM excitation, well above its TM reflection resonance frequency of 15.8 GHz.

Referring to the frequency range of interest, 0 to 5.0 GHz, the extracted parameters reveal the following. The effective wave impedance Z_eff_ shows that the MS itself is not matched to free space. As will be demonstrated with its integration with the driven array, it acts as a low loss impedance matching facilitator between the array and free space. The composite antenna and MS system are found to be well matched to free space over a wide bandwidth.

The extracted value of the effective permeability μ_eff_ is equal to one; i.e., the MS acts as a purely electric surface with no bianisotropic behaviour. The value of ε_eff_ changes by less than 4%, 8.545–8.882, over the entire frequency range of interest. These results further illustrate that by working well below the resonances, the lossy regions of the MS are avoided.

#### Measurements

**Figure 8:**
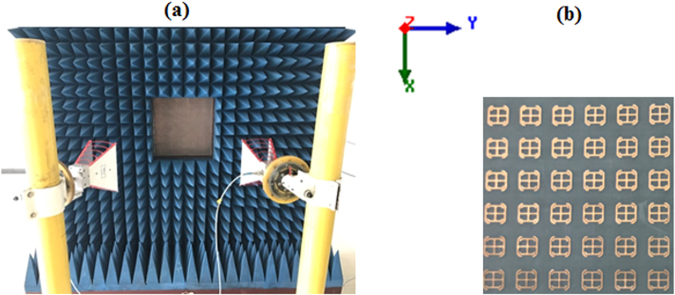
Measurement setup. (**a**) The reflection measurement and (**b**) an expanded view of the MS under test.

**Figure 9:**
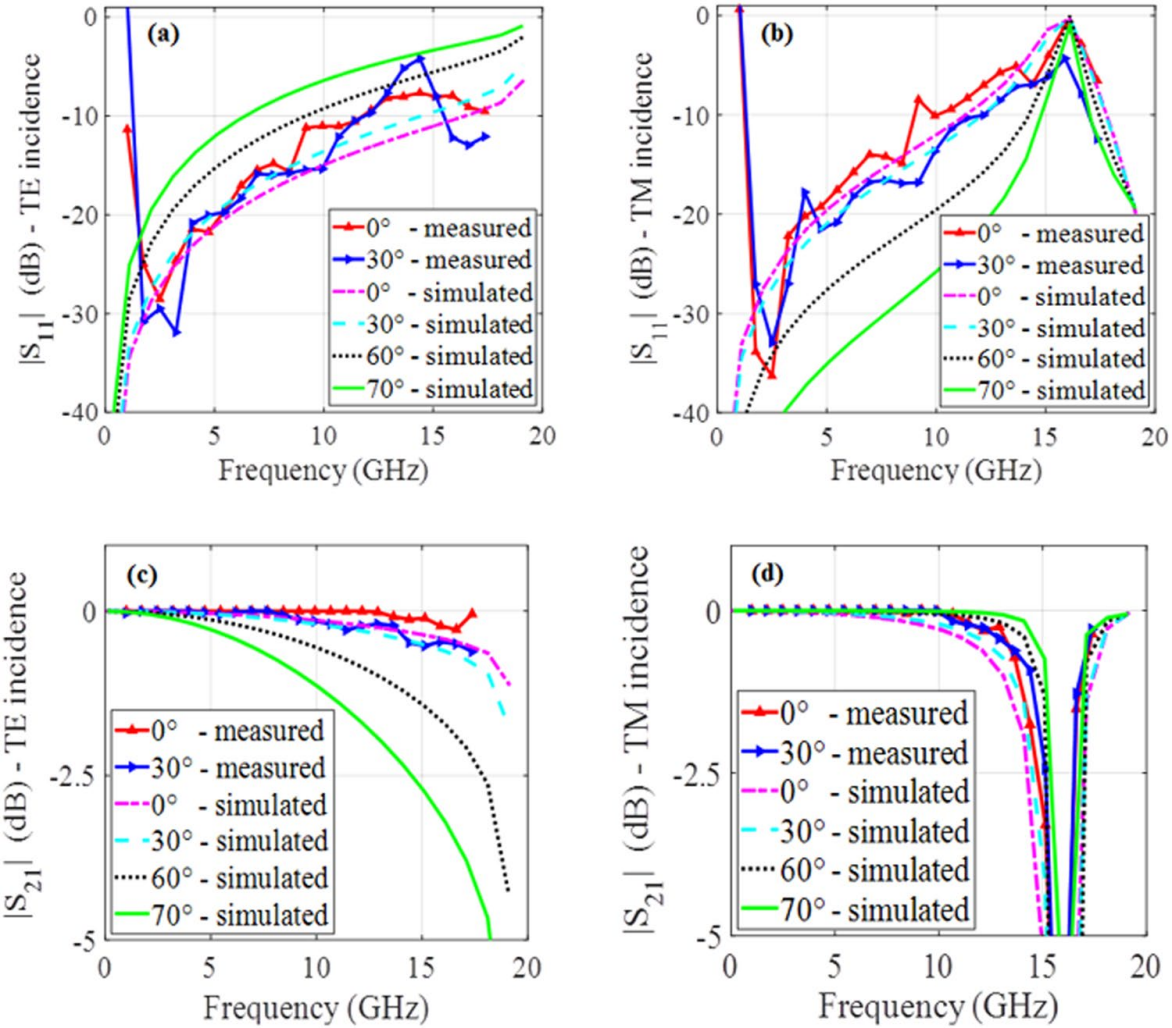
Simulated and measured transmission and reflection results for the MS. (**a**) Return loss - TE incidence. (**b**) Return loss - TM incidence. (**c**) Insertion loss - TE incidence. (**d**) Insertion loss - TM incidence.

To verify the simulation results, a 40×40 unit cell MS was fabricated and measured in an anechoic chamber. The test setup was in both reflection and transmission modes. As shown in Fig. 8(a), the reflection mode included two horn antennas for transmit and receive on the same side of the MS. An expanded view of the fabricated MS is also given in Fig. 8(b). The simulated and measured transmission and reflection results for the MS are shown in Fig. 9. It is noted that the measurements were carried out only up to 18.0 GHz again to due to the chamber limitations. Consequently, only the TM reflection resonance at 15.8 GHz could be verified experimentally. The simulated results show very good scanning up to 70° with acceptable transmission and reflection losses for both the TE and TM excitations. Measurements were carried out for broadside and for 30° incidence; those results show good agreement with the simulated results. For the reflection measurements, the simulated and measured results agree very well except below 3.0 GHz. These discrepancies have been determined to be mainly a consequence of the limited functional frequency range of the absorbers available in the anechoic chamber. The transmission measurements on the other hand, agree well with the simulated results across the whole measured band.

#### Metasurface integrated with a TCAA

In order to demonstrate the scanning advantages of including a MS with a driving antenna array, an infinite TCAA was simulated with both an infinite dielectric slab WAIM^31,32^ and then with an infinite version of the TC-UAJC based MS. The infinite nature of the geometries allowed us to simulate a unit cell of the corresponding finite TCAA-WAIM system. While this choice neglected some minor edge effects, it captures the essence of the performance of the realistic structure given the large extent of the fabricated MS sample. The TCAA is a wideband array and, hence, was selected to test appropriately the wideband performance of the MS. To analyze the reference dielectric slab WAIM, it was placed directly above the antenna array with the optimized parameters: the relative permittivity ε_r_ = 2.5 and thickness of dielectric slab h_di_ = 12.0 mm.

**Figure 10:**
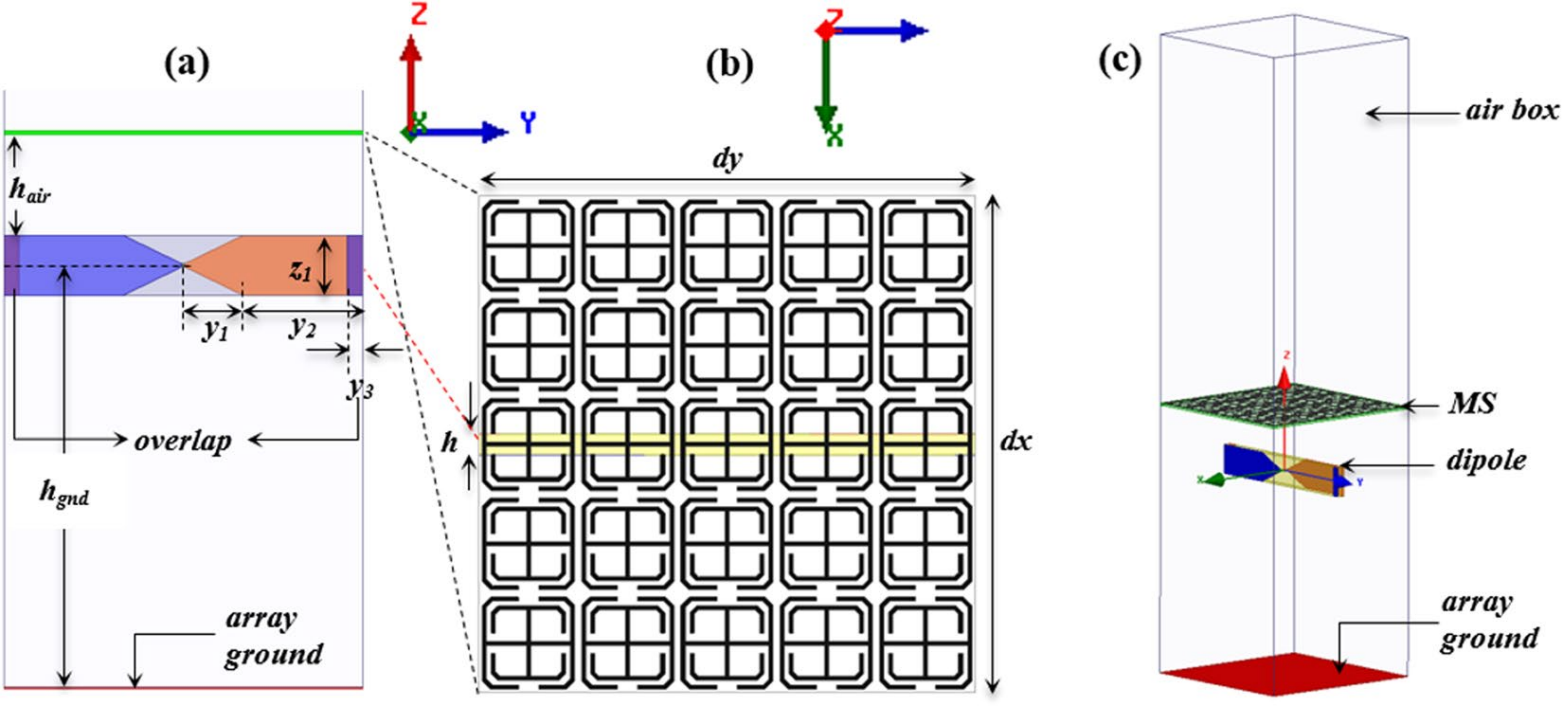
TCAA integrated with the MS. (**a**) Side view of a unit cell with vertically oriented dipole arms printed on opposite sides of the PCB. Blue = top layer, orange = bottom layer, purple = overlap with adjacent elements. (**b**) Expanded top view of the modified MS within one unit cell. The dimensions of the various parameters of the TCAA-WAIM unit cell are: z_1_ = y_1_ = 4.0 mm, y_2_ = 8.0 mm, y_3_ = 1.0 mm, h_air_ = 6.76 mm, h_gnd_ = 28.25 mm, h = 1.016 mm, and dx = dy = 24.0 mm. (**c**) Perspective view of the unit cell.

Figure 10(a) shows a side view of one unit cell of the TCAA integrated with the MS structure. The TCAA is made up of an infinite dipole array with overlapping arms and fed with lumped ports. It is oriented vertically along the *y-z* plane with arms printed on opposite sides of a thin printed circuit board (PCB) substrate. The blue colored arm of the dipole is on the top layer of the substrate and the orange colored arm is on its bottom layer. The purple region, indicated by *y*_3_, represents the amount of overlap with the arms of the adjacent elements. The arms were assumed to be perfect electric conductors (to significantly reduce the computational overhead) mounted on a Rogers RT/Duroid 6010 substrate with a dielectric constant of 10.2 and a thickness of 1.016 mm. Each element was driven with a lumped port whose active input impedance was (170 − j38) ${\rm{\Omega }}$. Figure 10(b) shows the top view of the MS which is made up of a 5×5 array of the TC-UAJC MS elements within the TCAA unit cell. The dimensions of the MS were adjusted to account for the interactions between it and the antenna. The new optimized dimensions were: r_1_ = 1.59 mm, g_1_ = 0.9774 mm, g_2_ = 0.2384 mm, L_2_ = 4.4568 mm. All other dimensions remained the same.

**Figure 11:**
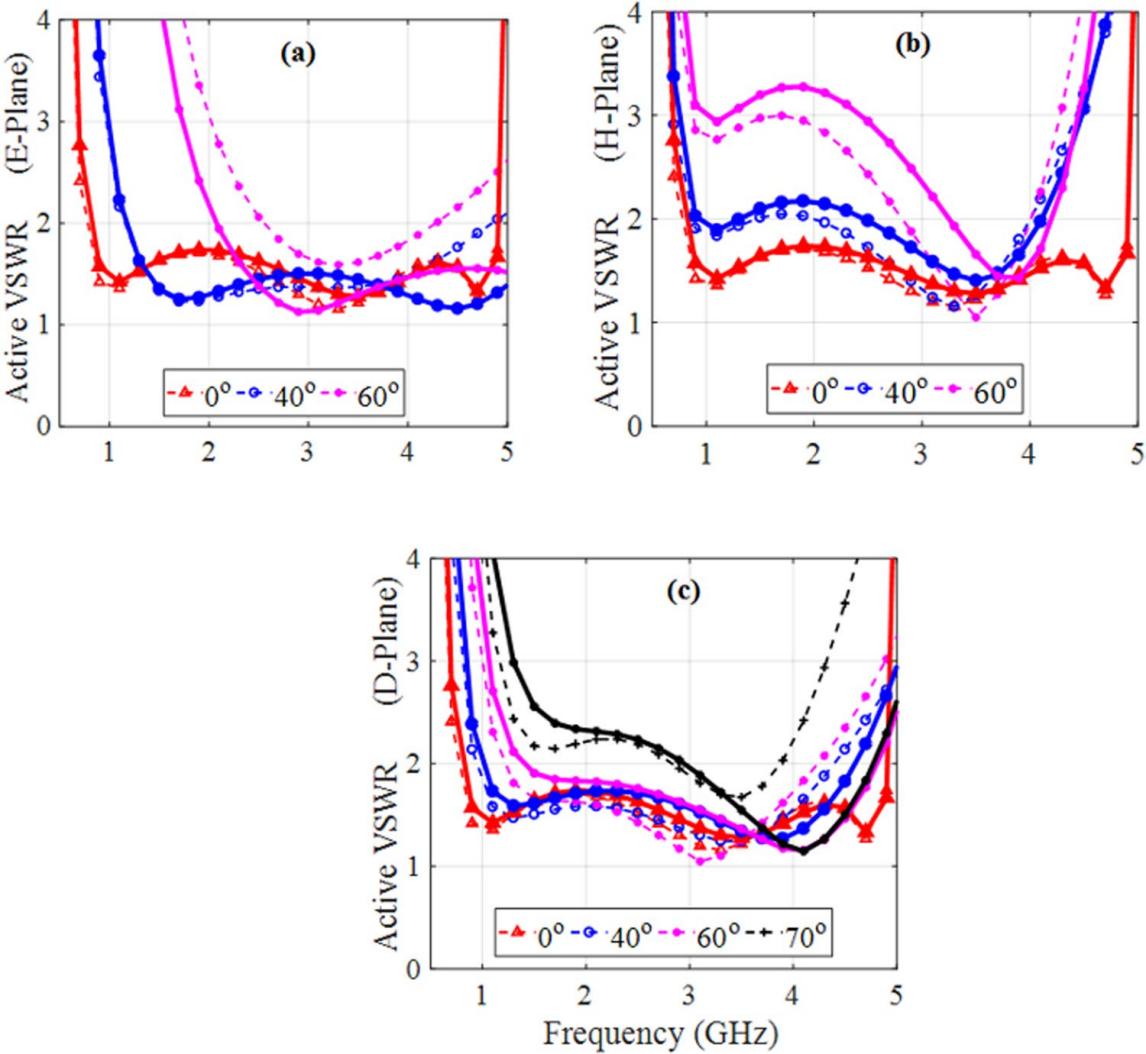
Scanning of the MS (solid lines) and dielectric WAIM (dashed lines) systems. (**a**) E-plane. (**b**) H-plane and (**c**) D-planes.

The scanning abilities of the MS and dielectric WAIM systems are compared in Fig. 11 across the E, H, and D (diagonal) planes over a wide bandwidth. The dashed lines represent the dielectric WAIM results and the solid lines represent the MS results. At broadside, the simulated performance characteristics of the TCAA integrated with the dielectric and MS WAIMs are virtually identical. However, the MS system has the added advantage of more degrees of freedom with the potential to provide a better impedance match to the assumed 50-Ω source. Across the E-Plane, both the MS and dielectric WAIMs can scan to 60°, however, the MS can do so with a lower VSWR at wider scan angles. Across the H- and D-Planes, the two systems have very similar performance with the dielectric slab system being slightly better over the lower frequency band while the MS performs better over the higher frequency band. Nevertheless, both the dielectric and MS-based WAIM systems scan well out to 70° across their D-planes. Moreover, it can be observed that the MS system covers a wider bandwidth for all scan angles. The MS-array combination (37.264 mm) is also of a lower profile compared to the dielectric array combination (42.25 mm).

**Figure 12:**
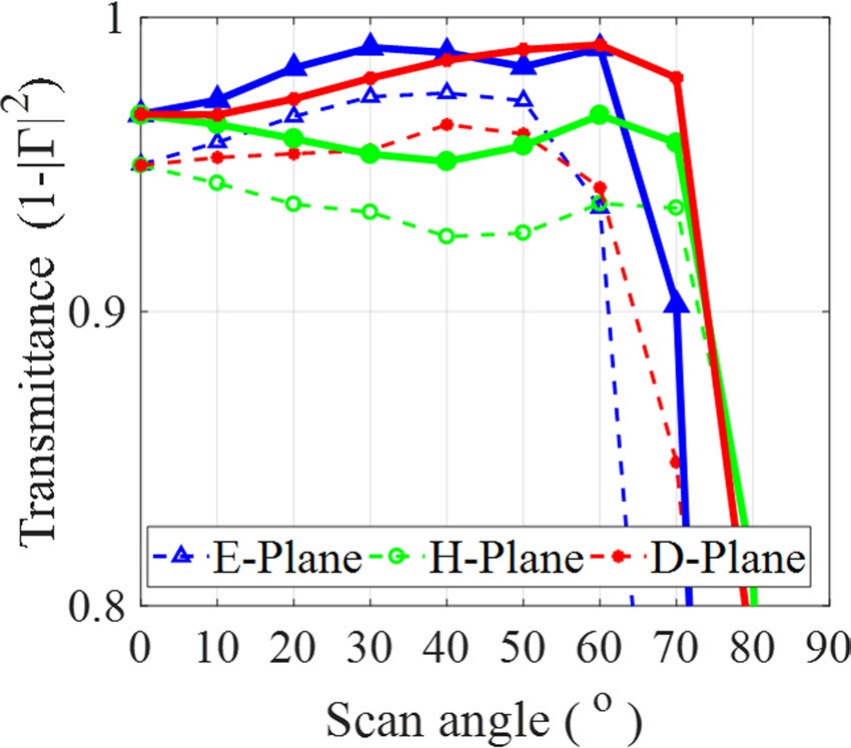
Comparison of the MS (solid lines) and dielectric WAIM (dashed lines) across the E, H, and D planes at a minimum of 80% transmittance (equivalent to VSWR < 3) at 4.0 GHz.

The transmittance values of the MS and dielectric WAIMs for all scan angles across the E, H, and D planes are compared in Fig. 12 at 4.0 GHz. It is interesting to find that the MS outperforms the dielectric WAIM across both the E and D planes and performs the same as the dielectric WAIM in the H plane. Previous works on single layer WAIMs based purely on the modification of the free space region above the array aperture are overwhelmingly carried out at a single frequency of operation. Therefore, to make a direct comparison to them, we compare in Table 1 the performance of the MS at 4.0 GHz to those reported in two key works in this area. The work in ref.^31^ only presents scanning across the H-plane for two designs. One scans up to 55° and another scans between 40° and 80°. It is shown here that the MS achieves wider scanning in both the E and H planes in comparison to the results reported in both ref.^31,32^. Moreover, it falls only 2° short of the D-plane scan in ref.^32^.

**Table 1:** Maximum Scan Range at a Single Frequency.

	Previous work		This work	
	Ref.^31^	Ref.^32^	Dielectric	MS
E-Plane	N/A	45°	64°	72°
H-Plane	55°	65°	80°	80°
D-Plane	N/A	81°	72°	79°

## Discussions

A wideband MS to be used as a WAIM layer has been systematically designed and its characteristics were experimentally validated. Its effective material parameters were extracted to show the constancy of ε and μ over the desired, very wide operational band of frequencies. The optimized MS was used as a superstrate over a TCAA of simple printed dipoles to reduce the impedance mismatch of the system to its source when the array is scanning. It was shown that the MS provides improved scanning along the E and D planes over its wide bandwidth without the use of bulky isotropic dielectrics or multi-layered structures. Furthermore, it maintained the scanning performance in the H plane. An MS-TCAA prototype integrated with a realistic feed network will be reported in the near future.

## Methods

The proposed MS was simulated and optimized in ANSYS-HFSS using master-slave periodic boundary conditions and two Floquet ports to illuminate both sides of the MS. The copper-cladded Rogers RT/Duroid^TM^ 5880 with a relative dielectric constant of 2.2 and a height of *H*_*sub*_ = 0.254 *mm* was selected as the substrate. The TC-UAJC elements were etched on the 17 μm thick copper sheet on the top surface of the dielectric substrate. To minimize the simulation time and the amount of discretization, the metals in the HFSS model were taken to be perfect electric conductors (PECs). To verify the MS simulation results, a 40×40 unit cell array was fabricated and measured in an anechoic chamber. Measurements were carried out using an Agilent vector network analyzer and two horn antennas to obtain the transmission and reflection coefficients for both TE and TM polarizations by rotating the horns through 90°. In the transmission case, the horn antennas are place equidistant on opposite sides of the MS and the signal received from the transmitting horn is measured for both broadside and 30° incidence. For the reflection measurements, the transmit and receive horns were on the same side of the MS as shown in Fig. 8. The effective medium parameters of the MS were extracted from the MSs S-parameters using MATLAB and the method described in ref.^50^.

The MS was integrated with a TCAA in an ANSYS-HFSS model to demonstrate its WAIM capabilities across a wide frequency range.
